# Genomic Insight Into the Population Structure and Admixture History of Tai-Kadai-Speaking Sui People in Southwest China

**DOI:** 10.3389/fgene.2021.735084

**Published:** 2021-09-20

**Authors:** Xiaoyun Bin, Rui Wang, Youyi Huang, Rongyao Wei, Kongyang Zhu, Xiaomin Yang, Hao Ma, Guanglin He, Jianxin Guo, Jing Zhao, Meiqing Yang, Jing Chen, Xianpeng Zhang, Le Tao, Yilan Liu, Xiufeng Huang, Chuan-Chao Wang

**Affiliations:** ^1^College of Basic Medical Sciences, Youjiang Medical University for Nationalities, Baise, China; ^2^State Key Laboratory of Cellular Stress Biology, School of Life Sciences, Xiamen University, Xiamen, China; ^3^Department of Anthropology and Ethnology, Institute of Anthropology, School of Sociology and Anthropology, National Institute for Data Science in Health and Medicine, Xiamen University, Xiamen, China; ^4^State Key Laboratory of Marine Environmental Science, Xiamen University, Xiamen, China; ^5^Department of Forensic Medicine, Guizhou Medical University, Guiyang, China; ^6^Jinzhou Medical University, Jinzhou, China

**Keywords:** genetic substructure, genetic admixture, population history, Tai-Kadai-speaking Sui people, East Asia

## Abstract

Sui people, which belong to the Tai-Kadai-speaking family, remain poorly characterized due to a lack of genome-wide data. To infer the fine-scale population genetic structure and putative genetic sources of the Sui people, we genotyped 498,655 genome-wide single-nucleotide polymorphisms (SNPs) using SNP arrays in 68 Sui individuals from seven indigenous populations in Guizhou province and Guangxi Zhuang Autonomous Region in Southwest China and co-analyzed with available East Asians *via* a series of population genetic methods including principal component analysis (PCA), ADMIXTURE, pairwise Fst genetic distance, *f*-statistics, *qpWave*, and *qpAdm*. Our results revealed that Guangxi and Guizhou Sui people showed a strong genetic affinity with populations from southern China and Southeast Asia, especially Tai-Kadai- and Hmong-Mien-speaking populations as well as ancient Iron Age Taiwan Hanben, Gongguan individuals supporting the hypothesis that Sui people came from southern China originally. The indigenous Tai-Kadai-related ancestry (represented by Li), Northern East Asian-related ancestry, and Hmong-Mien-related lineage contributed to the formation processes of the Sui people. We identified the genetic substructure within Sui groups: Guizhou Sui people were relatively homogeneous and possessed similar genetic profiles with neighboring Tai-Kadai-related populations, such as Maonan. While Sui people in Yizhou and Huanjiang of Guangxi might receive unique, additional gene flow from Hmong-Mien-speaking populations and Northern East Asians, respectively, after the divergence within other Sui populations. Sui people could be modeled as the admixture of ancient Yellow River Basin farmer-related ancestry (36.2–54.7%) and ancient coastal Southeast Asian-related ancestry (45.3–63.8%). We also identified the potential positive selection signals related to the disease susceptibility in Sui people *via* integrated haplotype score (iHS) and number of segregating sites by length (nSL) scores. These genomic findings provided new insights into the demographic history of Tai-Kadai-speaking Sui people and their interaction with neighboring populations in Southern China.

## Introduction

Southwest China is home to diverse ethnic minorities and linguistic families. Previous population genetic studies based on genetic markers including mitochondrial DNA (mtDNA) and Y-chromosome haplogroups, short tandem repeats (STR), insertion/deletion polymorphisms (InDels), and genome-wide single-nucleotide polymorphisms (SNPs) shed light on the genetic profile and demographic history of ethnolinguistic groups from southern China and Southeast Asia (Wen et al., 2004; Kutanan et al., 2017; Liu C. et al., 2018; He et al., 2020; Huang et al., 2020; Liu D. et al., 2020; Liu J. et al., 2020; Liu Y. et al., 2020; Wang C. C. et al., 2021). A SNP chip-based population study from the genomic perspective demonstrated that the genetic profile of Tai-Kadai-speaking Hainan Li from southernmost China (referred to as Hlai) was less affected by the Neolithic farming expansion or historical migration compared with other mainland Tai-Kadai-speaking populations; Hlai-related lineage contributed a large proportion of the ancestry to the mainland Tai-Kadai-speaking populations (He et al., 2020). Huang et al. (2020) found that the bidirectional gene flow between Tai-Kadai- and Hmong-Mien-speaking populations formed the Hmong-Mien/Tai-Kadai cline; Tai-Kadai-related groups also had a strong impact on the genetic makeup pattern of populations in Mainland Southeast Asia in the recent two millennia. From the ancient genomic perspective, Wang C. C. et al. (2021) reconstructed the deep population history of East Asia and found a kind of ancestry probably related to the Neolithic Yangtze River farmers, which contributed widely to present-day Austronesian speakers and Tai-Kadai speakers. Yang et al. (2020) revealed that Neolithic Fujian-related ancestry contributed substantially to the present-day Southern Chinese and Southeast Asians; during the Early Neolithic period, Northern East Asians related to Coastal Shandong-related ancestry migrated southward and shifted the genetic makeup of populations from southern China. Additionally, Wang T. et al. (2021) recently reported that Guangxi Longlin-related ancestry, Fujian Qihe-related ancestry, and deep East Asian Hoabinhian-related ancestry participated in the formation of Early Neolithic Guangxi ancients (represented by Dushan/Baojianshan) but limitedly contributed to present-day Southeast Asians; the historical Guangxi samples possessed a genetic profile similar to that of present-day Hmong-Mien- and Tai-Kadai speaking populations.

Our studied population, Tai-Kadai-speaking Sui people, is officially recognized as one of the 56 ethnic groups in China. The Chinese “Sui” means “Water,” reflecting the living environment and lifestyles of Sui people. More than 80% of Sui people inhabited Guizhou, one of the most ethnolinguistically diverse provinces in southwest China; the rest of the Sui resided in adjacent provinces in China, such as Guangxi, Yunnan, and Sichuan (2010 Census). According to the historical accounts, the ancestors of Tai-Kadai-speaking populations were ancient Baiyue tribes, the indigenous people living in southern China. Forced by warfare and famine during the Qin dynasty (circa second century B.C.), Chinese Han continuously expanded toward the south for a long time. A great many Baiyue people migrated to southwest China and then formed the Tai-Kadai-speaking people (Gao, 2002; Zhang and Zhang, 2018). Published genetic evidence invalidated the origin of Tai-Kadai-speaking Sui people. The maternally inherited mtDNA HVS-1 region analysis showed Sui had high frequencies of mtDNA haplogroups, which were dominant in southern China [B (B4a, 3.3%; B4b1, 6.7%; and B5a, 20%), M7 (M^∗^, 6.7%; M7b^∗^, 6.7%; M7b1, 6.7%; and M8a, 6.7%), F (F^∗^, 3.3%; F1a, 20%; and F3, 13.3%), and R (R9b, 3.3%)] (Li et al., 2007a). From the paternal Y-chromosome side, Li et al. investigated the haplotype network of Y-STRs, showing that the haplotype O1a-M119 was the dominating haplogroup in Tai-Kadai-speaking Sui (F, 8%; K, 10%; O1a^∗^, 18%; O2a^∗^, 44%; and O3a5, 20%), as well as in Taiwan aborigines, but not in other East Asians, indicating the Sui was native to southern China (Li et al., 2008).

Previous genetic findings were predominately based on autosomal/X/Y STRs, mainly aimed to evaluate the forensic characterization of STR markers and investigate the genetic relationships between the Guizhou Sui people and the surrounding Tai-Kadai-speaking, Hmong-Mien-speaking Miao, and Sinitic populations living in southern China (Yang et al., 2012; Ji et al., 2017; Chen et al., 2018; Guo et al., 2019). Thus, the fine-scale genetic structure and admixture history, the potential positive selection signals of Tai-Kadai-speaking Sui populations, especially Guangxi Sui, are still underrepresented owing to a lack of genome-wide data. In this study, we genotyped 498,655 SNPs of a total of 68 Sui individuals from seven populations in Southwest China and compared them with the published SNP dataset of present-day and ancient East Asians in order to advance the understanding of the demographic history of Sui people from a genomic perspective.

## Materials and Methods

### Sampling, Genotyping, and Quality Control

**FIGURE 1:**
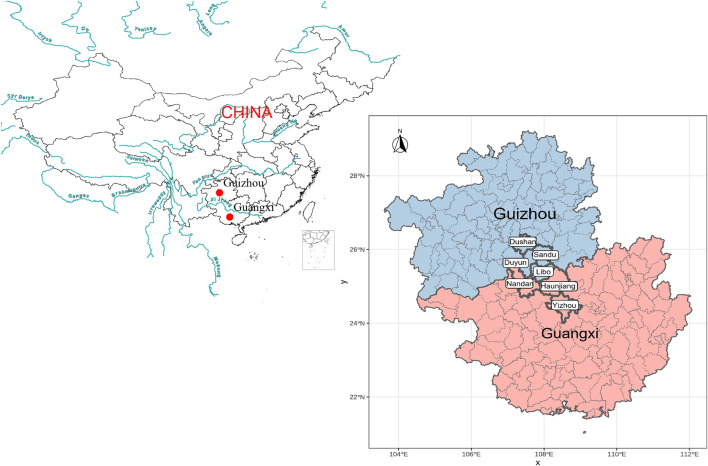
Geographical positions of new collected Sui people from Guangxi and Guizhou Province, Southwest China.

A total of 68 blood samples were collected from the seven populations from the Guangxi Zhuang Autonomous Region and Guizhou province with written informed consent and genotyped using Affymetrix WeGene V1 array. These samples were collected randomly from unrelated participants whose parents and grandparents are indigenous people and have a non-consanguineous marriage of the same ethnical group for at least three generations. The ethnicities of all participates were used as their self-declaration based on their family migration history and corresponding family records. Our study and sample collection were reviewed and approved by the Medical Ethics Committee of Youjiang Medical University for Nationalities and Xiamen University (approval number: XDYX2019009) and followed the recommendations provided by the revised Helsinki Declaration of 2000. After removing batch effects and missing sites, we genotyped 498,655 genome-wide SNPs. We listed the detailed sample information in Supplementary Table 1 and plotted the geographic sampling locations in Figure 1.

We further calculated the kinship coefficient *via* the GCTA software (Yang et al., 2011) using options “–autosome –make-grm” and then conducted PLINK1.9 (Purcell et al., 2007) with the option “–missing” to calculate the SNP calling rate for each individual and “–remove” to exclude the individuals with the lowest SNP calling rate and had up to third-degree kinship with other collected samples (kinship coefficient > 0.125). The genetic relationship matrix (GRM) was displayed in Supplementary Figure 1. Finally, we got 58 unrelated Sui individuals for further study.

### Merging Data

We merged our newly collected samples with published genome-wide SNP data of present-day and ancient East Asian and Southeast Asian populations (Patterson et al., 2012; Huang et al., 2018, 2020; Lipson et al., 2018; McColl et al., 2018; Liu Y. et al., 2020; Ning et al., 2020; Yang et al., 2020; Wang T. et al., 2021;^1^). Two datasets were used in subsequent population genetic analysis: (1) we merged our newly collected Sui individuals with a 1240K capture dataset to create the high-SNP-density “merged-1240K” dataset harboring 373,933 SNP sites; and (2) we merged our newly published Sui people data with 82 present-day populations or nine meta-populations and 40 ancient groups from the Affymetrix Human Origins (HO) panel to generate the “merged-HO” dataset, covering lower SNP sites (119,349) but maximum the number of populations and individuals. Data merging was done by *mergeit* from EIGENSOFT (Patterson et al., 2006).

### Principal Component Analysis

We carried out PCA on the “merged-HO” dataset by the *smartpca* program of EIGENSOFT (Patterson et al., 2006) with the default parameters and lspproject: YES. We only used modern populations to construct PCs and then projected the ancient samples onto the top two PCs.

### ADMIXTURE

Before the ADMIXTURE analyses, we pruned SNPs on the “merged-HO” dataset in strong linkage disequilibrium with each other using PLINK1.9 (Purcell et al., 2007) by parameters –indep-pairwise 200 25 0.4 and then ran ADMIXTURE (Alexander et al., 2009) with default parameters from *K* = 2 to 12. The cross-validation error reached the lowest point at *K* = 4 (Supplementary Figure 2).

### Pairwise Fst Genetic Distance

Modern populations which harbored genomic information of more than five individuals on the “merged-HO” dataset were used to calculate pairwise Fst following Weir and Cockerham (1984). We estimated Fst by *smartpca* using EIGENSOFT (Patterson et al., 2006) with default parameters and inbreed: YES and fsonly: YES. The neighbor-joining (N-J) phylogenetic relationship was constructed using Mega 7.0 (Kumar et al., 2016). Populations in the same clade or branches indicated that they had closer relationships than populations in different clades.

### *f3*-Statistics and *f4*-Statistics

We used the *qp3pop* and *qpDstat* packages implemented in ADMIXTOOLS (Patterson et al., 2012) with default parameters to calculate the *f*-statistics. Statistical significance was assessed using the default blocked jackknife approach implemented in ADMIXTOOLS. Outgroup-*f*_3_*(X, Y; outgroup)* calculated the shared genetic drift between X and Y since their divergence from the outgroup. *Admixture-f*_3_*(source1, source2; target)* evaluated the admixture signals in the targets (*Z*-score < −3). In the form of *f*_4_*(outgroup, W; X, Y)*, a *Z*-score > 3 implied that W shared more alleles with Y than with X; a *Z*-score less than −3 suggested that W shared extra alleles with X compared with Y; a | *Z*-score| < 3 indicated that X and Y formed a clade in relation to the outgroup and W. We used an African population Yoruba as the outgroup.

### Genetic Homogeneity Testing by *qpWave*

#### Pairwise *qpWave* Test

We used pairwise *qpWave* as implemented in ADMIXTOOLS (Patterson et al., 2012) on the “merged-1240K” dataset to test whether pairwise populations were genetically homogeneous in relation to a set of outgroups. We used Mbuti, Mongolia_N_East, DevilsCave_N, Ami, Liangdao2, and Vietnam_LN as outgroups because those groups were unlikely to have recent gene flow with our studied Sui people and might be differently related to the ancestral sources of Sui people. A *p*-value > 0.05 for rank = 0 suggested that pairwise populations were homogeneous genetically relative to outgroups. A *p*-value < 0.05 for rank = 0 indicated that a minimum of two streams of ancestry were needed to relate pairwise groups to the outgroups.

#### Outgroup-Dropping Pairwise *qpWave* Test

We did the “outgroup-dropping” test in which we dropped one of the populations in the outgroup set by turn to investigate which outgroups may lead to the nonhomogeneity between the pairwise-tested populations.

### Admixture Coefficient Modeling by *qpAdm*

We used *qpAdm* as implemented in ADMIXTOOLS (Patterson et al., 2012) with default parameters and all snps: YES to estimate admixture proportions for one target population as a combination of N-specified source populations by exploiting the shared genetic drift with a set of outgroups. The models with a *p*-value > 0.05, nested *p*-value < 0.05, and admixture proportions estimated between (0, 1) were accepted.

#### Two-Way Admixture Model

We used ancient Northern East Asian-related ancestry (represented by YR_LN) and ancient Southeast Asian-related ancestry (represented by Liangdao2) as sources. Seven populations (Mbuti, Tianyuan, Papuan, Onge, DevilsCave_N, Japan_Jomon, and Mongolia_N_East) were used as outgroups.

#### Three-Way Admixture Model

We further used YR_LN, Ami, Vietnam_N as proxies for ancient Northern East Asian, ancient coastal Southeast Asian, and ancient inland Southeast Asian-related ancestries. Nine populations (Mbuti, Tianyuan, Papuan, Onge, Liangdao2, DevilsCave_N, Japan_Jomon, Mongolia_N_East, and Malaysia_LN) were used as outgroups.

### Detecting the Positive Natural Selection Signals

Before identifying the natural selection, we used PLINK1.9 (Purcell et al., 2007) to remove individuals whose SNP-missing-rate is greater than 10% with parameter –geno 0.1 and then applied ShapeIT with default parameters (Delaneau et al., 2013) to phase autosomal SNP data of the Sui people. We calculated the integrated haplotype score (iHS) (Voight et al., 2006) and number of segregating sites by length (nSL) (Ferrer-Admetlla et al., 2014) for each phased SNP site (377,197) *via* the selscan software with default parameters (Szpiech and Hernandez, 2014). Then we used selscan’s norm package to normalize the scores within every 100 bins of allele frequency. A total of 1,829 SNPs with both absolute normalized iHS and an nSL greater than the threshold (top 1% iHS: 2.566430; top 1% nSL: 2.533570) were regarded as the candidate sites under natural selection. We then perform (1) the gene annotation *via* 3DSNP (Lu et al., 2016) and (2) KEGG analysis *via* Kobas (Bu et al., 2021).

## Results

### Investigating the Population Structure of Studied Sui Populations

**FIGURE 2:**
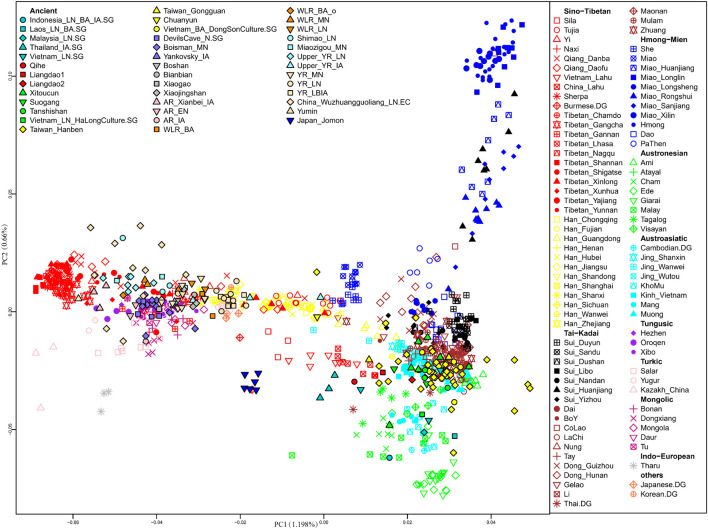
Principal component analysis (PCA) was constructed based on our studied Sui groups and published present-day East Asian and Southeast Asian populations, then projected ancient East Asians onto the first two principal components.

We first carried out PCA to uncover an overview of the genetic structure of East Asians and Southeast Asians (Figure 2). The top two PCs (PC1 and PC2) revealed that within present-day populations, individuals from the same linguistic classification and geographic locations were mostly placed together, displayed as the following genetic clusters: Altaic-related (Tungusic speaking, Turkic speaking, and Mongolic speaking); Tibetan-Burman-related; Japanese- and Korean-related; Han-related North-South cline; and Southeast Asian-related clusters which comprised Hmong-Mien-related, Austronesian-related, Austroasiatic-related, and Tai-Kadai-related populations.

The distributions of the studied Sui people were relatively scattered, with two major clusters: one genetic cline consisting of Huanjiang Sui individuals from Guangxi, which was placed on the Hmong-Mien-related cline; and the other comprising the rest of the newly studied Sui groups, clustered together with published Tai-Kadai-speaking populations and displaying closer genetic relationships with AA, AN, and Sinitic-speaking populations from Southern China and Southeast Asia. Yizhou Sui from Guangxi slightly shifted toward the Han-related cline. Furthermore, we detected that the Sui-related cluster tended to deviate to HM-related cline compared with other published TK speakers; Tibetan-Burman-related and Altaic-related groups exhibited significant genetic differentiation with the studied Sui people. After projecting the ancient EA and SEA individuals onto the top two PCs, we observed that most ancient individuals fall relatively close with geographically close modern populations. Coastal Fujian_EN (Liangdao1, Liangdao2, and Qihe), Fujian_LN (Xitoucun, Suogang, and Tanshishan), Taiwan_IA (Taiwan_Hanben and Taiwan_Gongguan), Inland Vietnam Bronze Age (Vietnam_BA_DongSonCulture) individuals plotted relatively close with our studied Sui populations than other published ancient samples.

**FIGURE 3:**
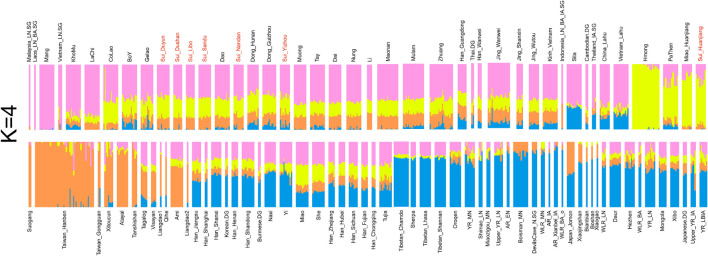
Model-based ADMIXTURE results showed the individual ancestry component composition of studied Sui and reference East Asian and Southeast Asian individuals with the predefined four ancestral sources (*K* = 4).

Model-based ADMIXTURE with an optimal K of 4 (Figure 3) suggested that four major EA and SEA ancestral components can adequately explain the genetic makeup of the studied populations: (1) The Inland Southeast Asian-related component (noted as pink) was maximized in Late Neolithic ancient SEAs from Vietnam, Malaysia, Laos, and modern Austroasiatic speakers Mang from Vietnam. This lineage also reached high proportions in the populations from southern China and Southeast Asia, such as Tai-Kadai speakers Dai and Austroasiatic speakers Jing. (2) The Hmong-Mien-related ancestry (denoted as yellow) was dominant in Hmong from Vietnam and Huanjiang_Miao from southern China. (3) The coastal Southeast Asian-related ancestry (denoted as orange) was enriched in the Neolithic Fujian populations, Iron Age Gongguan, and Hanben individuals from Taiwan as well as present-day indigenous Austronesian-speaking Ami and Atayal. (4) The Northern East Asian-related (denoted as blue) ancestry maximized in Tibetans and was also widely distributed in Sinitic and Altaic speakers and ancient Northeast Asians. The ADMIXTURE model in *K* = 4 revealed the difference in genetic compositions within Sui populations. The studied Huanjiang Sui individuals were characterized by a considerable amount of Hmong-Mien-related ancestry (∼70%), with ∼22% Inland Southeast Asian-related ancestry, ∼7% coastal Southeast Asian-related ancestry, and very few Northern East Asian-related ancestry (less than 1%). While the primary ancestry component assigned to other studied Sui groups as well as neighboring TK and AA speakers was the Inland southeast Asian-related component, they harbored less Hmong-Mien-related ancestry but higher proportions of coastal Southeast Asian-related and Northern East Asian-related ancestry compared with Huanjiang Sui. In addition, Yizhou Sui harbored a significantly higher Northern East Asian-related component than each Sui population (Yizhou Sui: mean = 8.5% versus Sui_Dushan: mean = 1.43%; Sui_Duyun: mean = 1.9%; Sui_Huanjiang: mean = 0.28%; Sui_Libo: mean = 0.56%; Sui_Nandan: mean = 2.79%; Sui_Sandu: mean = 0.88%; *p* < 0.02102, Student’s *t*-test).

### Population Relationships Between Studied Sui Groups and Worldwide Reference Populations

To explore the genetic affinity between the studied Sui and reference populations, we first constructed the unrooted N-J phylogenetic tree based on Wright’s fixation index pairwise Fst genetic distance among 79 modern populations (Supplementary Figure 3). We identified two main genetic branches highly correlated to the geographic locations; the sub-clades also corresponded well to the linguistic classifications: (1) the Northern East Asian-related one, which was made up of the Altaic-, Tibetan- Burman-, and Sinitic-speaking populations; and (2) the Southeast Asian-related cluster composed of the HM-, TK-, AN-, and AA-speaking populations. The Sui people showed a relatively close phylogenetic relationship with neighboring Tai-Kadai-speaking populations, such as Maonan, Dong, and CoLao. We observed the strong genetic assimilation within Sui groups except for Huanjiang Sui, which first clustered with HM speakers (Huanjiang Miao, Hmong, PaThen, and Hunan Miao) and then with TK-speaking CoLao and Dong, followed by other studied Sui populations.

**FIGURE 4:**
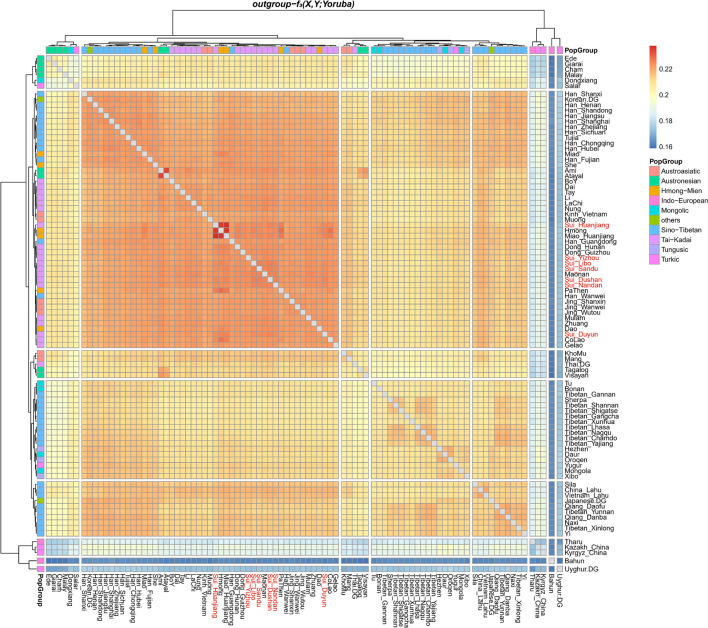
Heatmap showed the sharing genetic drift between the present-day East Asians estimated *via outgroup-f*_3_ *(modern East Asian 1, modern East Asian2; Yoruba)*. Red indicated more shared genetic drift among the pairwise populations since their divergence from an African outgroup Yoruba.

**FIGURE 5:**
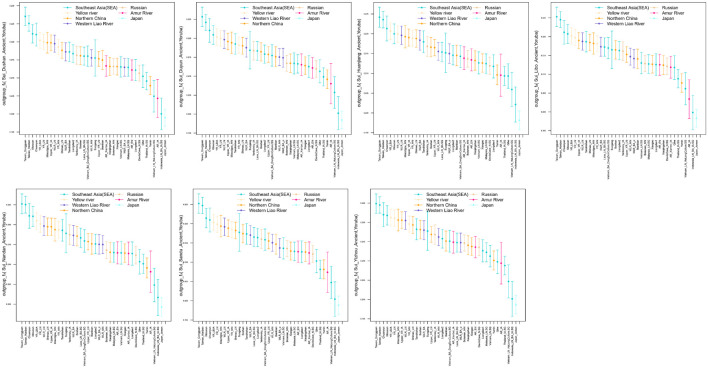
Error bar plot showed the genetic affinity between studied Sui populations and ancient East Asians revealed by *outgroup-f*_3_ *(ancient reference East Asians, Studied Sui; Yoruba)*. The error bar is marked as the standard deviation.

We subsequently conducted outgroup-*f*_3_-statistics in the form of *f*_3_*(X, Studied Sui; Yoruba)* to measure the shared genetic drift. The cluster patterns in the heatmap (Figure 4) confirmed that the studied Sui had a striking genetic affinity with each other and with present-day populations from southern China and Southeast Asia, especially Tai-Kadai speakers Maonan and Dong, Hmong-Mien speaker Hmong, and Austroasiatic speaker Jing. Huanjiang Sui individuals shared the highest genetic drift with Huanjiang Miao (*f*_3_: 0.237811), followed by Hmong (*f*_3_: 0.235770) and then by other Sui people (*f*_3_: 0.222949–0.225170). Focusing on the results of *outgroup-f*_3_*(Studied Sui, ancient individuals; Yoruba)* (Figure 5), we found that each Sui group displayed similar patterns of genetic affinity with ancient reference populations. The top highest shared drift with the studied Sui was provided by Iron Age Hanben and Gongguan samples from Taiwan (*f*_3_ > 0.223575), followed by Fujian Historical Chuanyun individuals (*f*_3_ > 0.220979), Fujian_LN Xitoucun individuals (*f*_3_ > 0.220718), and inland YR basin farmer populations (*f*_3_ > 0.215268).

To quantitatively evaluate the genetic similarity and differentiation among the studied Sui populations compared with the worldwide reference populations, we performed symmetrical *f*_4_-statistics in the form of *f*_4_*(Yoruba, X; Sui population 1, Sui population 2)* shown in Supplementary Table 2. The observed significant negative *f*_4_ values with absolute *Z*-scores larger than 3 indicated that X shared more genetic drift with *Sui population 1* relative to *Sui population 2*; otherwise, significant positive *f*_4_ values indicated more shared alleles between X and *Sui population 2* rather than *Sui population 1*. *Z*-scores ranging from (−3, 3) denoted that Sui 1 and Sui 2 formed one clade in relation to X and outgroup Yoruba, respectively. The observed significant *Z*-scores in *f*_4_*(Yoruba, X; Sui_Huanjiang, Sui population 2)* suggested that HM-speaking Hmong, Miao_Huanjiang, and PaThen shared excess alleles with Huanjiang Sui individuals (−18.139 ≤ *Z*-scores ≤ −4.691). Tai-Kadai-speaking Li, Mulam, Nung, and Tay and AA-speaking Kinh and Muong shared fewer alleles with Huanjiang Sui than with other Sui people (1.138 ≤ *Z*-scores ≤ 3.738). The significant negative *Z*-scores in *f*_4_*(Yoruba, Northern East Asian; Sui_Yizhou, Sui population 2)* indicated that there are significantly more derived alleles shared between Northern East Asians (such as Iron Age Amur River Basin-related_Xianbei populations, coastal Siberia Boisman_MN, DevilsCave_N, and Tibetan_Shannan) and Guangxi Yizhou Sui compared with Sui_Libo (*Z*-scores = −3.143, −3.045), Sui_Dushan (*Z*-scores = −3.085), and Sui_Duyun (*Z*-scores = −3.000). AN-speaking Ede shared excess alleles with Sui_Duyun (*Z*-scores = 3.101) and Sui Libo (*Z*-scores = 3.048) compared with Sui Yizhou. HM-speaking Hmong shared more derived alleles with Sui_Duyun than with Sui_Yizhou (*Z*-scores = 3.355). We did not identify the genetic difference among Guizhou Sui groups and Guangxi Sui_Sandu as no *f*_4_*(Yoruba, X; Guizhou Sui population 1, Guizhou Sui population 2/Sui_Sandu)* with significant *Z*-scores were observed.

**FIGURE 6:**
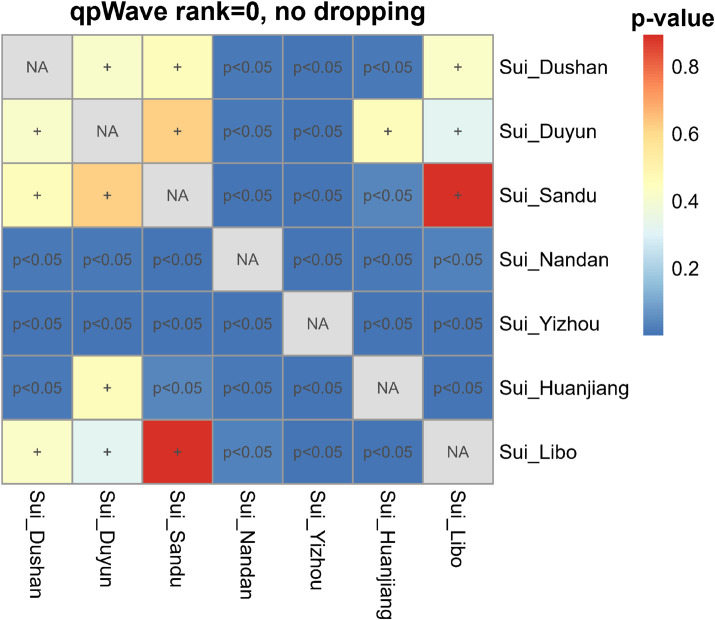
Testing the genetic homogeneity of pairwise Sui populations. Heatmap showed *p*-values (rank = 0) of pairwise *qpWave* in Guizhou and Guangxi Sui populations. The *p*-value > 0.05 was noted as +, indicating this pair of studied Sui groups derived from a single homogeneous population relative to a set of outgroups.

We then performed pairwise *qpWave* analysis, which was more accurate than *symmetry f*_4_-*statistics*, to further test whether seven Sui groups were genetically homogeneous (Figure 6). Each pair of Guizhou Sui populations had a *p*-value > 0.05 for rank = 0. We observed *p*-values < 0.05 (rank = 0) in the one-way admixture model when Guangxi Nandan, Huanjiang, and Yizhou Sui were used as one of the test populations. Pairwise *qpWave* results confirmed that (1) there is genetic homogeneity within Guizhou Sui individuals; (2) there is genetic heterogeneity within Guangxi Sui individuals; and (3) Guizhou Sui and Guangxi Sui were not derived from a single homogenous population in relation to the outgroups we used.

We next did the outgroup-dropping pairwise *qpWave* test (Supplementary Figure 4). Pairwise test populations which had genetic heterogeneity relative to the full set of outgroups showed a *p*-value > 0.05 (rank = 0) in the “outgroup-dropping pairwise *qpWave* test*”* instead, suggesting that the “dropped outgroup”-related ancestry might have a unique gene flow with one of the test groups, explaining the nonhomogeneity between the pairwise test populations. The results suggested that different levels of Ami-related gene flow may lead to the heterogeneity between Guangxi Sui and Guizhou Sui; coastal Amur River Neolithic DevilsCave-related ancestry may drive the nonhomogeneity between Yizhou Sui and other Sui groups.

### The Ancestry Inference of the Studied Sui People

We performed all possible *f*_4_-statistics in the form of *f*_4_*(Yoruba, X; studied group, Y)* for each Sui group to explore the possible extra gene flow that Sui people/Y received from X after the divergence between the specific population Y and studied Sui.

**FIGURE 7:**
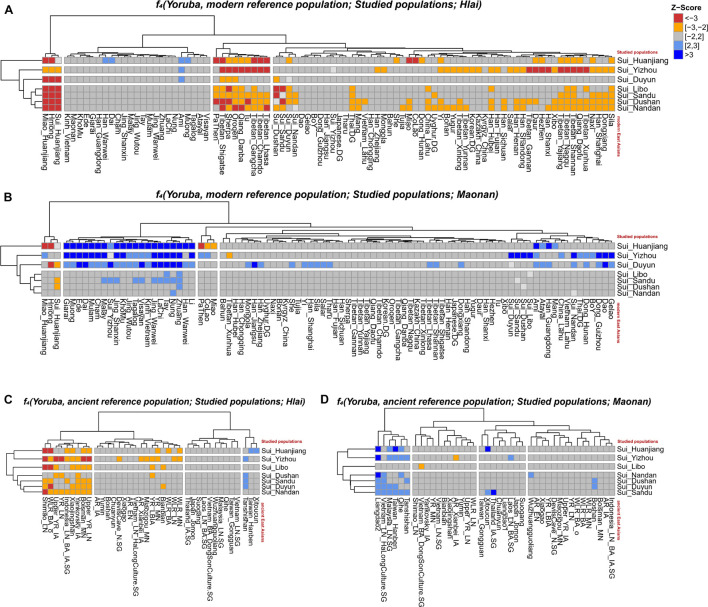
A series of *f*_4_ statistics performed in the form of **(A)** *f*_4_(Yoruba, modern EAs; studied groups; Hlai); **(B)** *f*_4_(Yoruba, modern EAs; studied groups, Maonan); **(C)** *f*_4_(Yoruba, ancient EAs; studied groups; Hlai); **(D)** *f*_4_(Yoruba, ancient EAs; studied groups, Maonan) to explore the genetic similarities and differentiation between studied Sui groups and Tai-Kadai speaking Hlai/Maonan.

When Tai-Kadai-speaking Hlai (representing the unadmixed form of Tai-Kadai-speaking populations) was same as Y (Figures 7A,C), the observed significant negative *Z*-scores suggested that Hmong-Mien speakers (Hmong and Miao_Huanjiang, −17.174 ≤ *Z*-scores ≤ −2.139) and Tibetan-Burman-related populations (such as Sherpa, −4.103 ≤ *Z*-scores ≤ −2.120) shared more derived alleles with our newly reported Sui people, indicating that the Sui people received the extra Hmong-Mien-related ancestry and northern East Asian sources after the separation of the Hlai and Sui groups or explaining that the Hlai received the ancestry which was a deeper linage than the common ancestor of Hmong-Mien-speaking populations and Sui people. No significant positive *f*_4_ values were observed.

As shown by the Fst-based N-J tree and *outgroup-f*_3_ statistics, the geographically close Tai-Kadai-speaking Maonan firstly clustered with Sui groups. Thus, we further did the formal test in the form of *f*_4_*(Yoruba, reference population; Sui, Maonan)* to explore the fine-scale genetic differentiation between the studied Sui and Maonan (Figures 7B,D, respectively). We observed that Guizhou Sui_Libo, Sui_Sandu, Sui_Dushan, and Guangxi Sui_Nandan had similar genetic profiles with Maonan (all | *Z*-scores| < 3). Hmong-Mien-speaking Miao groups from Vietnam and Guangxi showed additional gene flow with Guangxi Sui_Huanjiang (−17.884 ≤ *Z*-scores ≤ −17.838). Hmong shared more alleles with Sui_Duyun than with Maonan (*Z*-score = −3.044). Some of the Tai-Kadai speakers (such as Dai, Zhuang, Nung, Tay, and Lachi), Austroasiatic speakers (such as Jing), and Austronesian speakers (such as Ede) shared more alleles with Maonan than with the Sui_Huanjiang/Sui_Yizhou/Sui_Duyun (*Z*-scores ≥ 3.027). When X = ancient EA and SEA individuals, we observed that ancient Fujian_EN (Liangdao2) individuals shared more genetic drift with Maonan than with each newly studied Guangxi Sui (i.e., Sui_Huanjiang, Sui_Yizhou, and Sui_Nandan, *Z*-scores ≥ 3.611).

We exhausted all possible pairs of reference populations as genetic sources to estimate admixture signals in each studied Sui population *via admixture-f*_3_-*statistics*. The statistically significant negative *f*_3_ values with *Z*-scores less than −3 suggested that the target population might be an admixture of two source-related populations. Here, we reported the top 10 lowest *f*_3_ value results for each Sui group in the form of *f*_3_*(modern source1, modern source2; Studied Sui)* and *f*_3_*(ancient source1, ancient source2; Studied Sui)*, respectively, in Supplementary Tables 3, 4.

When focused on *f*_3_*(modern source1, modern source2; Studied Sui)*, only three significant admixture signals were observed in Sui_Duyun when we used Hmong-Mien ancestry (Hmong) as one source and TK-related (Li/Mulam) or AA-related ancestry (Muong) as the other source (*Z*-scores ≤ −3.093). Although no more significant negative *f*_3_ values were identified when we used other Sui groups as targets, it is important to note that a nonnegative *admixture-f*_3_ value does *not* prove that there is no admixture. The lowest *admixture-f*_3_ values were achieved in each studied Sui group except Yizhou Sui when we used the same pairs of source populations as follows: (1) Hmong-Mien-related Miao_Huanjiang/Hmong as *source1* and Tai-Kadai related Li/Mulam/Gelao as *source2*; and (2) Hmong-Mien-related Miao_Huanjiang/Hmong as *source1* and AN related Ami/Atayal as *source2*. For each newly studied Guizhou Sui group (i.e., Sui_Dushan, Sui_Duyun, Sui_Sandu, and Sui_Libo), when we used Hmong-Mien-related Miao_Huanjiang/Hmong as *source1* and AA-related Muong as *source 2*, low *f*_3_ values can be produced. For Sui_Yizhou, when one of the source populations represented Sino-Tibetan-related ancestry (Sherpa/Tibetan_Shigatse) and the other represented TK-related (Li) or AN-related ancestry (Atayal), top negative *f*_3_ values were generated.

When focused on the form *f*_3_*(ancient ref1, ancient ref2; Sui)*, the negative *f*_3_ values were observed in all Sui groups when one source was from ancient Southeast Asia (such as inland Indonesia_LN_BA_IA, Vietnam_LN_HaLongCulture, and coastal Liangdao) and the other from ancient Northern East Asia (such as Miaozigou_MN and AR_IA), suggesting the north–south admixture pattern for Sui people.

**FIGURE 8:**
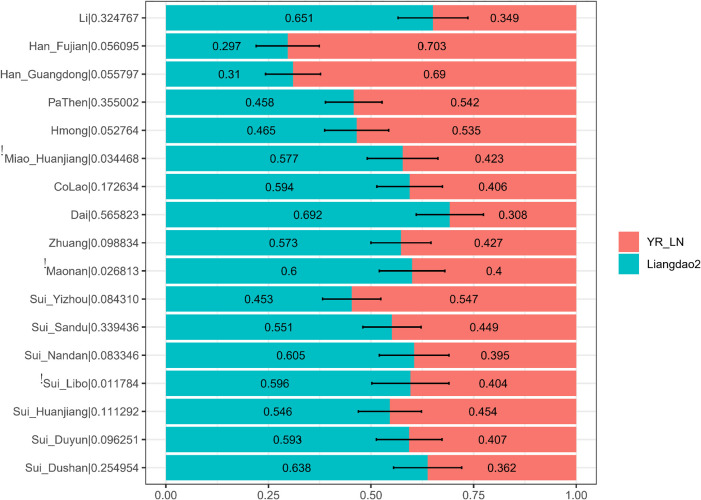
Ancestry mixture coefficients in the two-way admixture model *via qpAdm.* We used Liangdao2, YR_LN as proxies for ancient Southerneast Asian, ancient North East Asian, respectively. Mbuti, Tianyuan, Papuan, Onge, DevilsCave_N, Japan_Jomon, Mongolia_N_East were used as outgroups. For each target population, we reported the *p*-value for rank = 1 behind the corresponding population name in the form of “target name| *p*-value.” The population failed in *qpAdm* modeling (i.e., *p*-value < 0.05 for rank = 1) was noted as “!”. Error bar denoted the standard error estimated using jackknife.

We further applied *qpAdm* to explore the plausible admixture models for our studied Sui populations. We used the Late Neolithic Yellow River Basin farmer-related population (YR_LN) and Early Neolithic Coastal Liangdao2 individuals as proxies for the Northern East Asian-related and Southeast Asian-related source populations in a two-way admixture model (Figure 8). We observed that our newly studied Sui individuals were estimated to have 45.3–63.8% ancient Fujian Liangdao2 hunter-gatherer-related ancestry and 36.2–54.7% Yellow River farmer-related ancestry. The proportions of Liangdao2-related ancestry in our newly reported Sui_Huanjiang and Sui_Sandu individuals were at the same level, 54.6% (std.error = 7.7%) and 55.1% (std.error = 7.1%), respectively. Tai-Kadai-speaking Dai (69.2%, std.error = 8.2%) and Li (65.1%, std.error = 8.5%) and newly reported Sui_Dushan (63.8%, std.error = 8.3%), Sui_Duyun (59.3%, std.error = 8%), Sui_Libo (59.6%, *p*-value < 0.05, std.error = 9.4%), and Sui_Nandan (60.5%, std.error = 7.1%) samples harbored a similar proportion of Liangdao2-related ancestry. The Yizhou Sui had the highest proportion of YR_LN-related ancestry (54.7%, std.error = 7.1%) among Sui groups.

**FIGURE 9:**
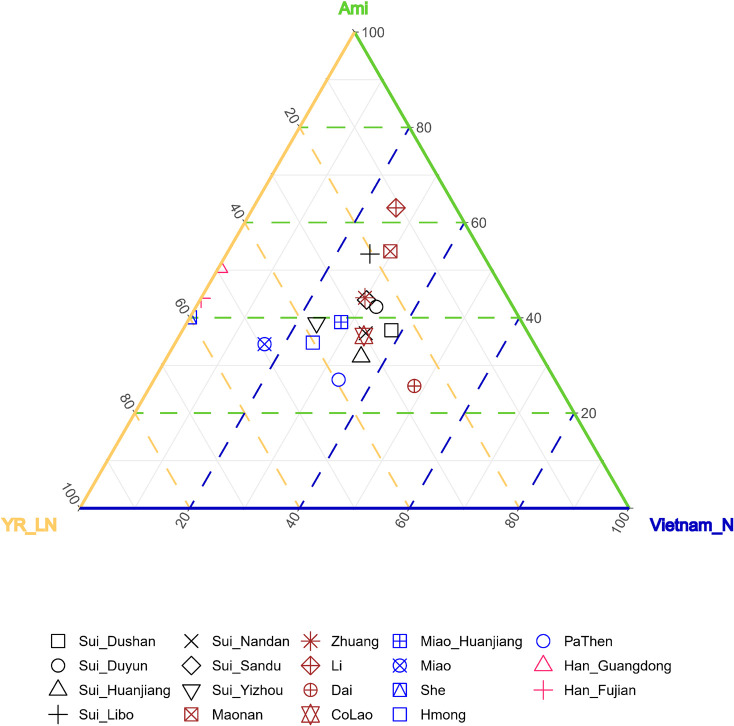
Ancestry mixture coefficients in the three-way admixture model *via qpAdm*. We used Inland Southeast Asian (represented by Vietnam_N), Coastal Southeast Asian (represented by Ami) and North East Asian (represented by YR_LN) as three proxies of the possible ancestral sources of studied Sui. Mbuti, Tianyuan, Papuan, Onge, Liangdao2, DevilsCave_N, Japan_Jomon, Mongolia_N_East, Malaysia_LN were used as outgroups. The Nested *p*-value > 0.05 when Han_Fujian, Han_Guangdong and She were used as target, suggesting it may be appropriate to drop Vietnam_N source from the model, so we finally showed the admixture proportions in two-way admixture model for these three groups in this figure.

As the results of *admixture-f*_3_-*statistics* suggested, our studied Sui might be modeled as an admixture of one ancient inland Southeast Asian group and one ancient North East Asian group. Therefore, we used coastal Southeast Asian (represented by Ami, the indigenous AN-speaking Taiwanese), inland Southeast Asian (represented by Vietnam_N), and Northern East Asian (represented by YR_LN) as three proxies of the possible ancestral sources to infer ancestry mixture coefficients in a three-way admixture model. The studied Sui groups covered similar proportions of coastal SEA ancestry and inland SEA ancestry and clustered with neighboring HM-speaking populations and TK-speaking populations in the ternary diagram (Figure 9).

### Detecting the Positive Natural Selection Signals of Sui People

**FIGURE 10:**
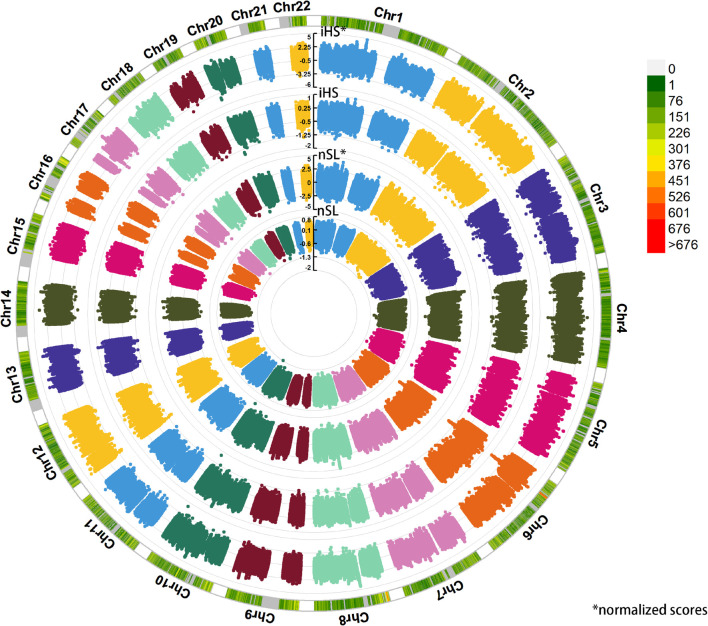
Detecting the positive selection signals. Here, we displayed the raw iHS scores, standardized iHS scores, raw nSL scores, standardized nSL scores *via* circle Manhattan plot.

We applied haplotype-based iHS and nSL statistical indexes to explore the putative positive selection signals in Sui people (Figure 10). We got 317,569 phased SNPs of 58 Sui individuals. A total of 1,829 candidate SNPs with (1) normalized iHS score > top 1% score and (2) normalized nSL score > top 1% score were then used for KEGG pathway analysis. We listed the KEGG enrichment results with a *p*-value < 0.05 and corrected *p*-value < 0.05 in Supplementary Figure 5. Candidate loci were mainly enriched in the pathway with regard to the susceptibility of complex diseases, cellular processes, and so on. Specifically, the SNP which had the highest iHS and nSL scores was SNP rs11599686 (chr10:123863188), located in the *TACC2* gene associated with the susceptibility of complex diseases such as breast cancer (Onodera et al., 2016).

## Discussion

The strong correlation between the population structure and linguistic classifications/geographic locations in East Asia has been reported in several genome-wide SNP-based studies (He et al., 2020; Huang et al., 2020; Kutanan et al., 2021; Wang C. C. et al., 2021). The population expansion with the extensive gene flow among populations which belong to the different linguistic classifications also drives the formation of the complex population genetic structure in East Asia (Huang et al., 2020; Liu D. et al., 2020; Kutanan et al., 2021; Wang M. et al., 2021; Wang C. C. et al., 2021; Yang et al., 2020). Yang et al. (2020) recently reconstructed the genetic structure and admixture history of Neolithic ancient NEAs and SEAs, demonstrating that the population structure in East Asia had existed early in the Neolithic; the spread of the NEA-related ancestry led to more genetic homogeneity in present-day EAs than in Neolithic EAs. Wang C. C. et al. (2021) demonstrated that the population expansion of the hunter-gatherers in Mongolia and Amur River Basin, Yellow River Basin farmers, Yangtze River Basin farmers, and Yamnaya Steppe pastoralists during the Holocene contributed to the formation of the population genetic structure in East Asia. Liu D. et al. (2020) analyzed the allele sharing and haplotype sharing profiles in present-day populations from five major language families in Vietnam and found extensive genetic interactions between Hmong-Mien-speaking populations and Tai-Kadai-speaking populations, such as the Tai-Kadai-speaking CoLao, who harbored more Hmong-Mien-related ancestry (represented by Hmong) compared with neighboring Tai-Kadai speakers.

The genetic profile of Tai-Kadai-speaking Sui people and the admixture history and genetic affinity with neighboring populations were largely unknown due to a lack of high-density sampling and genome-wide data. In this study, we comprehensively co-analyzed our newly genotyped genome-wide SNP data of 24 Guangxi Sui and 34 Guizhou Sui individuals with published ancient and present-day East Asians to elucidate (1) the genetic relationships between the Sui and reference East Asians; (2) the fine-scale population structure within the Sui people; (3) the admixture history of each Sui population; and (4) the potential positive selection signals of Sui people. The patterns of shared genetic drift measured *via outgroup-f*_3_-*statistics* and the phylogenetic relationships in Fst-based N-J tree supported the finding that the studied Sui had closer genetic relationships with Neolithic-to-modern populations from Southern China and Southeast Asians, especially present-day TK, HM, Sinitic, AA, and AN speakers and ancient Coastal Southeast Asians which were represented by Iron Age Taiwan Hanben and Gongguan individuals compared with most NEAs, supporting the hypotheses from the genomic perspective that Sui people were originally from southern China.

These results were in accordance with the genetic findings based on forensic-related genetic markers (X-STR, Y-STR, and autosomal-STR) indicating that the Sui people displayed a close affinity with geographically close populations, especially Qiandongnan Miao (Chen et al., 2018), AA-speaking Jing (Yang et al., 2012), and Maonan and Guizhou Han (Guo et al., 2019).

Results of PCA and model-based ADMIXTURE analysis revealed the genetic substructure within seven studied Sui populations: (1) the TK-speaking Sui individuals from Guangxi Huanjiang formed a cline which was localized on the intermediate of Hmong-Mien-related genetic cline, rather than clustered with neighboring TK-speaking populations, and (2) a relatively loose cluster which consisted of the rest of the newly reported Sui populations, partially overlapped with TK-speaking populations from Southern China and Southeast Asia.

Symmetric *f*_4_-*statistics* and pairwise *qpWave* consistently showed that the Guizhou Sui people were relatively homogeneous and showed similar genetic profiles with Tai-Kadai-related populations, such as Maonan. While Guangxi Sui groups were relatively heterogeneous, we observed excessive genetic affinity between Tai-Kadai-speaking Huanjiang Sui and the geographically close Hmong-Mien-speaking Miao people although the two groups were ethnically different. Huanjiang Sui did not share the most derived alleles with other Guizhou and Guangxi Sui people, which belonged to the same ethnic group, suggesting that Huanjiang Sui received a significantly geographically close Hmong-Mien-speaking Miao-related ancestry (represented by Huanjiang Miao) after Huanjiang Sui and other Sui people separated from the common ancestor. Sino-Tibetan-related populations contributed to the extra ancestry to Yizhou Sui people compared with other Sui groups, indicating that the Yizhou Sui have been primarily affected genetically by the surrounding Han populations. These results suggested the differentiated demographic history among the studied Sui populations.

The significant negative *Z*-scores of *f*_4_*(Yoruba, East Asians; studied Sui, TK-speaking Li)* revealed that the ancestors of the Sui people might experience excessive admixture events with HM and NAEs after the divergence with the unmixed, indigenous TK-speaking proxy Li islanders. The significant negative values in *admixture-f*_3_*(Hmong, Li/Mulam; studied Sui_Duyun)* suggested that the ancestor of HM-related populations might also participate in the formation of the Sui people. Hmong-Mien-speaking populations, such as Miao and Yao, are the dominant ethnic groups in southwest China. While the Sui has a relatively small population, there is only one autonomous country for Sui people in China (i.e., Sandu Autonomous Country of Guizhou). Previous studies suggested that Hmong-Mien-related people might be the direct descendants of Daxi-related people as there was a rare Y-chromosome haplogroup O3d detected in Neolithic Yangtze river Daxi culture-related people and modern Hmong-Mien speakers (Su et al., 1999; Li et al., 2007b). A possible scenario is that Hmong-Mien-speaking populations carried more Neolithic Yangtze River farmer-related ancestry and had a distinct genetic profile when compared with Sui people; the ancestor of the Sui people migrated southward (according to the historical documents) admixed with the indigenous Hmong-Mien-related populations, transforming the genetic makeup of Sui populations. More Yangtze River-related ancient samples in Neolithic, Bronze Age, Iron Age, and Historical Age are expected to be studied. The weak admixture signals in *admixture-f*_3_ *statistics* shed light on the potential north–south admixture patterns for the studied Sui groups. Furthermore, the *qpAdm*-based admixture model demonstrated that Sui people could be modeled as the admixture of ancient Northern East Asians (ANEAs) and ancient southeast Asians (ASEAs), in which ANEAs were represented by Neolithic Yellow River Basin-related farmer populations and ASEAs were characterized by Neolithic/modern Coastal Southern East Asians. More specifically, in the three-way admixture model, Sui people and neighboring HM- and TK-speaking populations derived their ancestry from more similar (but still different) proportions of ANEA-, coastal SEA-, and inland SEA-related components compared with the Han from Southern China. Conclusively, the formation of the population structure of Tai-Kadai-speaking populations might be plausibly explained by (1) the differential proportions of the primary ancient sources and (2) the various levels of gene flow with surrounding people, such as HM-speaking and Sinitic-speaking populations.

Finally, we detected the potential positive selection signals based on the phased data of Sui people *via* normalized iHS and nSL. Notably, lots of significant SNPs enriched in the KEGG pathway associated with the susceptibility of complex diseases (such as the Type 1 diabetes mellitus pathway and gastric cancer pathway). When focused on the SNPs which were famous for the natural selection in East Asians in previous genetic studies (Sabeti et al., 2007; Peng et al., 2010; Calandra et al., 2011; Kamberov et al., 2013), we observed SNPs rs3827760 (chr2:109513600) on the *EDAR* gene (associated with hair thickness and facial morphology), rs4148211 (chr2:44071742) on the *ABCG8* gene (associated with lipid metabolism), and rs1229984 (chr4:100239318) located in the *ADH1B* gene (associated with alcohol metabolism), which were likely under natural selection in Sui people (rs3827760: normalized iHS score = 2.788630; normalized nSL score = no results; rs4148211: normalized iHS score = 2.536000; normalized nSL score = 1.980440; rs1229984: normalized iHS score = 2.096340; normalized nSL score = 2.303840).

However, more population genetic studies based on the whole-genome sequence (WGS) data *via* next-generation sequencing (NGS) and single-molecule real-time sequencing (SMRTS) of Sui individuals from more geographic locations (such as Yunnan, Sichuan, and Jiangsu) are expected to be conducted to provide genomic insight into the formation of Sui people and to dissect the complex demographic history of populations from Southern China and Southeast Asia comprehensively.

## Data Availability Statement

The datasets presented in this study can be found in online repositories. The names of the repository/repositories and accession number(s) can be found below: https://zenodo.org/record/5483577, doi: 10.5281/zenodo.5483577.

## Ethics Statement

The studies involving human participants were reviewed and approved by Medical Ethics Committee of Youjiang Medical University for Nationalities and Xiamen University (Approval Number: XDYX2019009). The patients/participants provided their written informed consent to participate in this study.

## Author Contributions

C-CW and XH designed the study. RuW and C-CW wrote the manuscript. XB, YH, RoW, and XH collected the samples. KZ, XY, HM, GH, JG, JZ, MY, JC, XZ, LT, and YL conducted the experiment. RuW and C-CW analyzed the data. All authors reviewed the manuscript.

## Conflict of Interest

The authors declare that the research was conducted in the absence of any commercial or financial relationships that could be construed as a potential conflict of interest. The reviewer SW declared a past co-authorship with the authors XY, GH, JG, JZ, and C-CW to the handling editor.

## Publisher’s Note

All claims expressed in this article are solely those of the authors and do not necessarily represent those of their affiliated organizations, or those of the publisher, the editors and the reviewers. Any product that may be evaluated in this article, or claim that may be made by its manufacturer, is not guaranteed or endorsed by the publisher.

## Footnotes

1
https://reich.hms.harvard.edu/downloadable-genotypes-present-day-and-ancient-dna-data-compiled-published-papers

## Supplementary Material

Supplementary Figure 1 The Genetic Relationship Matrix (GRM). **(A)** based on the raw data (68 Sui individuals); **(B)** based on the clean data which removed the kinship (58 Sui individuals).

Supplementary Figure 2 Cross-Validation error for model-based ADMIXTURE analysis. The lowest cross validation error occurred at *K* = 4.

Supplementary Figure 3 Neighbor-joining tree based on Fst genetic distance among studied Sui and present-day EA and SEA individuals.

Supplementary Figure 4 *qpWave* Outgroup dropping test, in which we dropped one of the populations in the outgroup set by turn (Mbuti, Mongolia_N_East, DevilsCave_N, Ami, Liangdao2, Vietnam_LN). If we observed *P* > 0.05 for rank = 0, while *P* < 0.05 (rank = 0) in the no-drop qpWave test, suggesting the dropped population might have a unique gene flow with one of the test groups, explained the non-homogeneity between the pairwise test populations.

Supplementary Figure 5 KEGG pathway analysis. Each row represented an enriched function, and the length of the bar represented the enrich ratio which was calculated as “input gene number”/“background gene number.” The color of the bar represents different clusters.

## Abbreviations

SNP: single-nucleotide polymorphism
HO: Human Origin
PC: principal component
N: Neolithic
EN: Early Neolithic
MN: Middle Neolithic
LN: Late Neolithic
BA: Bronze Age
IA: Iron Age
H: Historical
SEA: Southeast Asia
EA: East Asia
TK: Tai-Kadai
AN: Austronesian
AA: Austroasiatic
HM: Hmong-Mien
YR: Yellow River.
