# Supplementary material for: Genomic Insight Into the Population Structure and Admixture History of Tai-Kadai-Speaking Sui People in Southwest China
Source: Front Genet. 2021 Sep 20;12:735084. doi: 10.3389/fgene.2021.735084 (PMC8489805; doi:10.3389/fgene.2021.735084)
Supplement: Supplementary Figure 1 — The Genetic Relationship Matrix (GRM). (A) based on the raw data (68 Sui individuals); (B) based on the clean data which removed the kinship (58 Sui individuals). [file Data_Sheet_1.pdf]

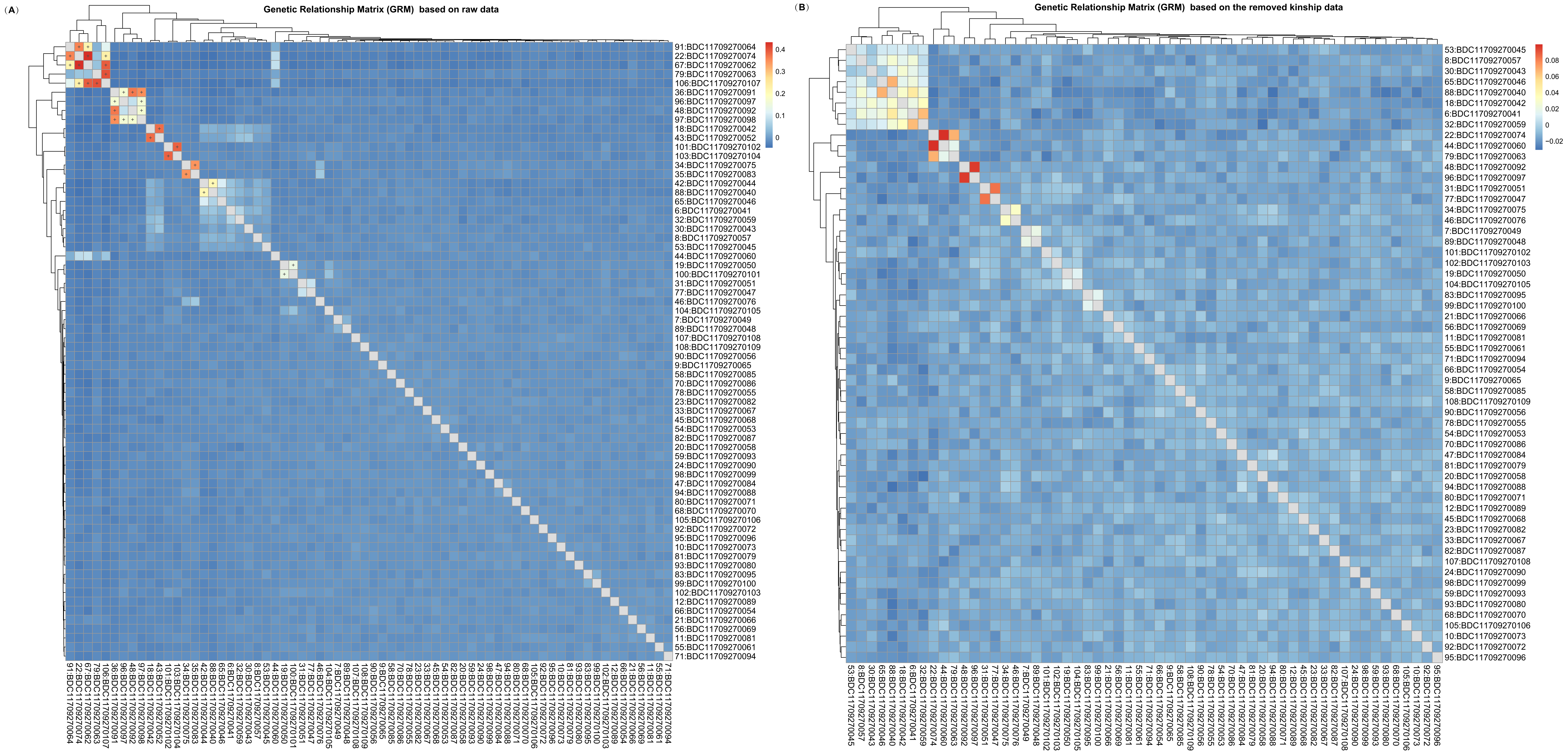
(A)
Genetic Relationship Matrix (GRM) based on raw data
0.4
0.3
0.2
0.1
0
91:BDC11709270064
22:BDC11709270074
67:BDC11709270062
79:BDC11709270063
106:BDC11709270107
36:BDC11709270091
96:BDC11709270097
48:BDC11709270092
97:BDC11709270098
18:BDC11709270042
43:BDC11709270052
101:BDC11709270102
103:BDC11709270104
34:BDC11709270075
35:BDC11709270083
42:BDC11709270044
88:BDC11709270040
65:BDC11709270046
6:BDC11709270041
32:BDC11709270059
30:BDC11709270043
8:BDC11709270057
53:BDC11709270045
44:BDC11709270060
19:BDC11709270050
100:BDC11709270101
31:BDC11709270051
77:BDC11709270047
46:BDC11709270076
104:BDC11709270105
7:BDC11709270049
89:BDC11709270048
107:BDC11709270108
108:BDC11709270109
90:BDC11709270056
9:BDC11709270065
58:BDC11709270085
70:BDC11709270086
78:BDC11709270055
23:BDC11709270082
33:BDC11709270067
45:BDC11709270068
54:BDC11709270053
82:BDC11709270087
20:BDC11709270058
59:BDC11709270093
24:BDC11709270090
98:BDC11709270099
47:BDC11709270084
94:BDC11709270088
80:BDC11709270071
68:BDC11709270070
105:BDC11709270106
92:BDC11709270072
95:BDC11709270096
10:BDC11709270073
81:BDC11709270079
93:BDC11709270080
83:BDC11709270095
99:BDC11709270100
102:BDC11709270103
12:BDC11709270089
66:BDC11709270054
21:BDC11709270066
56:BDC11709270069
11:BDC11709270081
55:BDC11709270061
71:BDC11709270094
(B)
Genetic Relationship Matrix (GRM) based on the removed kinship data
0.08
0.06
0.04
0.02
0
−0.02
53:BDC11709270045
8:BDC11709270057
30:BDC11709270043
65:BDC11709270046
88:BDC11709270040
18:BDC11709270042
6:BDC11709270041
32:BDC11709270059
22:BDC11709270074
44:BDC11709270060
79:BDC11709270063
48:BDC11709270092
96:BDC11709270097
31:BDC11709270051
77:BDC11709270047
34:BDC11709270075
46:BDC11709270076
7:BDC11709270049
89:BDC11709270048
101:BDC11709270102
102:BDC11709270103
19:BDC11709270050
104:BDC11709270105
83:BDC11709270095
99:BDC11709270100
21:BDC11709270066
56:BDC11709270069
11:BDC11709270081
55:BDC11709270061
71:BDC11709270094
66:BDC11709270054
9:BDC11709270065
58:BDC11709270085
108:BDC11709270109
90:BDC11709270056
78:BDC11709270055
54:BDC11709270053
70:BDC11709270086
47:BDC11709270084
81:BDC11709270079
20:BDC11709270058
94:BDC11709270088
80:BDC11709270071
12:BDC11709270089
45:BDC11709270068
23:BDC11709270082
33:BDC11709270067
82:BDC11709270087
107:BDC11709270108
24:BDC11709270090
98:BDC11709270099
59:BDC11709270093
93:BDC11709270080
68:BDC11709270070
105:BDC11709270106
10:BDC11709270073
92:BDC11709270072
95:BDC11709270096
