# Supplementary figures and images for: Genomic Insight Into the Population Structure and Admixture History of Tai-Kadai-Speaking Sui People in Southwest China
Source: Front Genet. 2021 Sep 20;12:735084. doi: 10.3389/fgene.2021.735084 (PMC8489805; doi:10.3389/fgene.2021.735084)

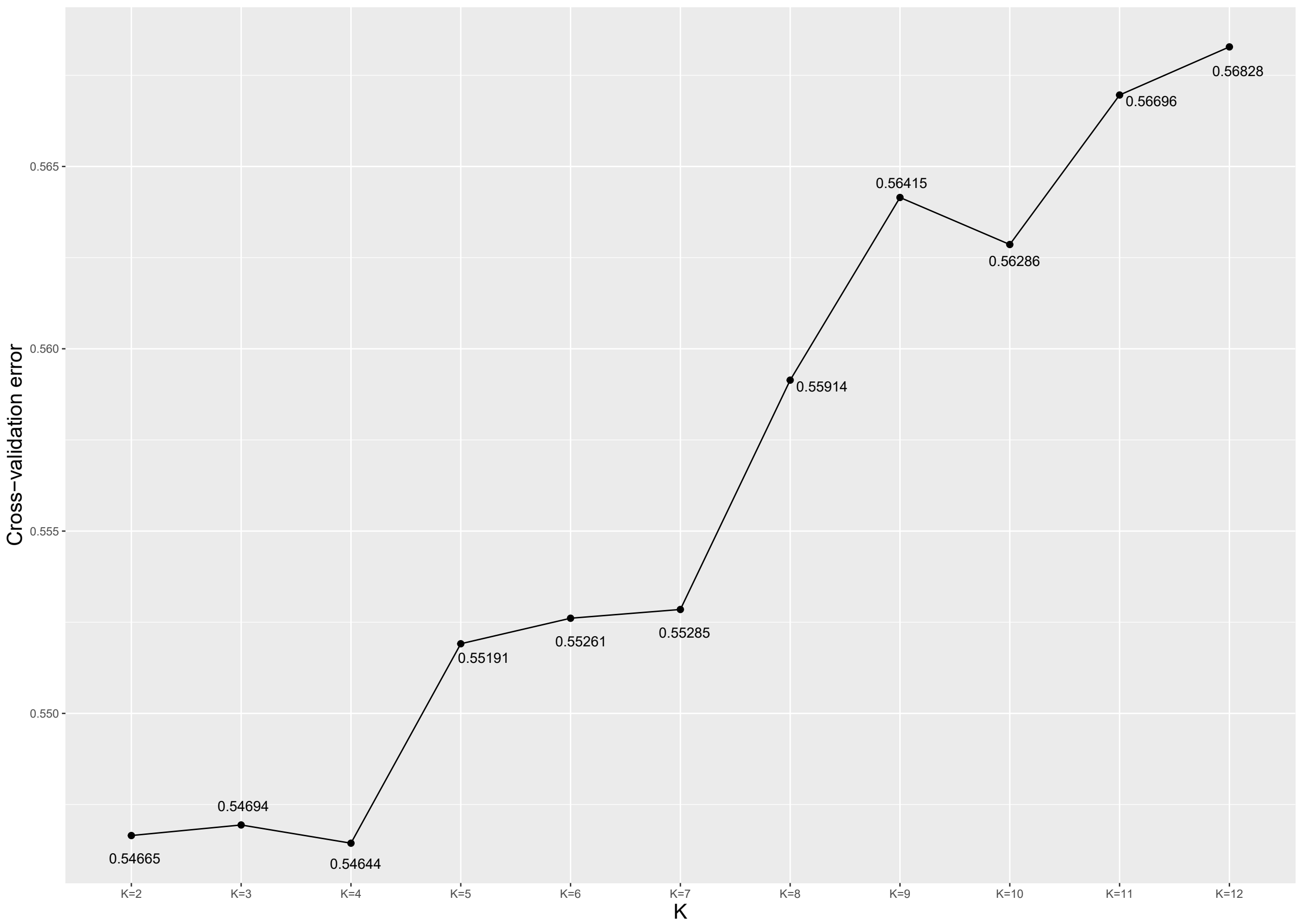

Supplement: Supplementary Figure 2 — Cross-Validation error for model-based ADMIXTURE analysis. The lowest cross validation error occurred at K = 4. [file Data_Sheet_2.pdf]

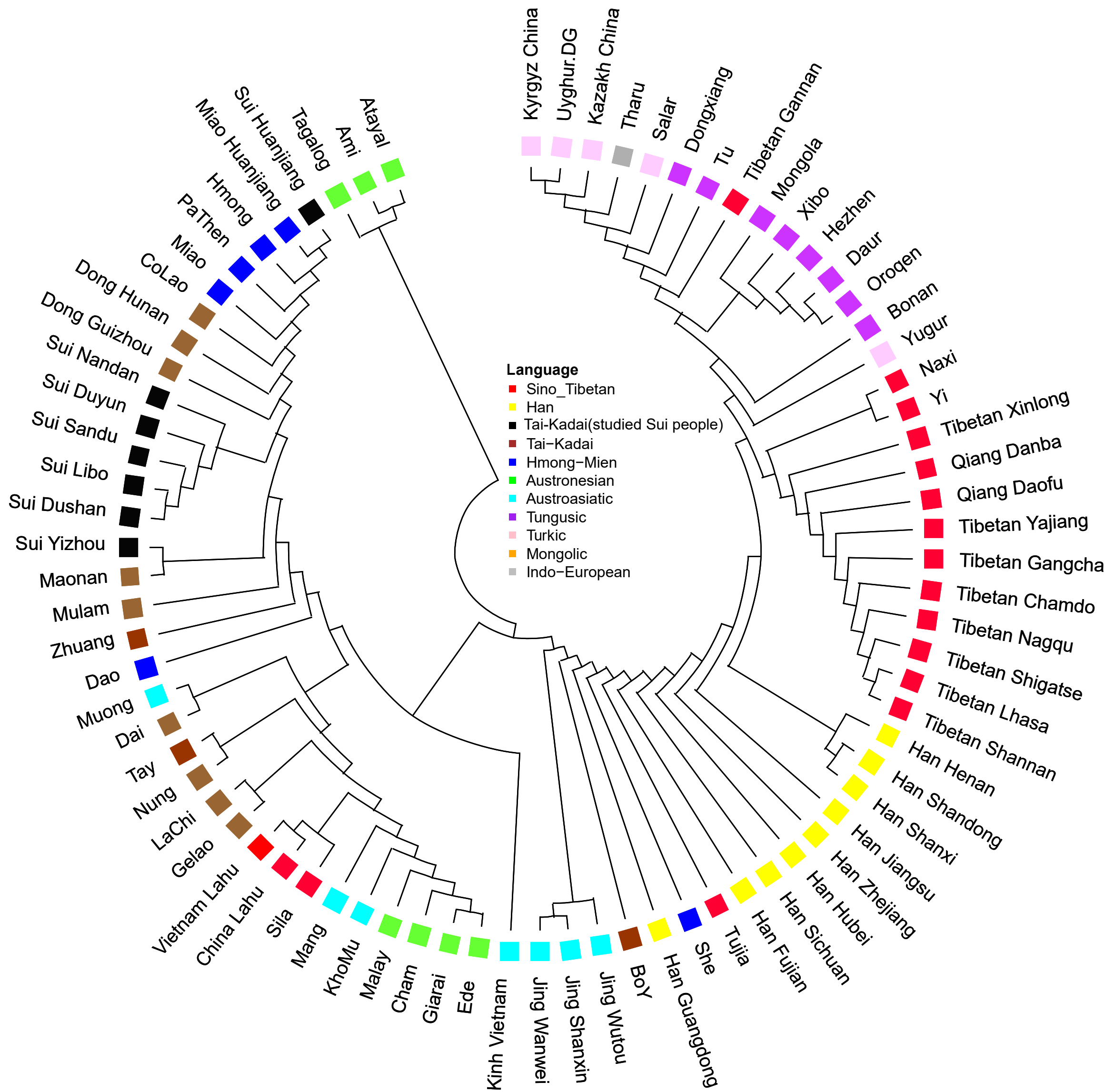

Supplement: Supplementary Figure 3 — Neighbor-joining tree based on Fst genetic distance among studied Sui and present-day EA and SEA individuals. [file Data_Sheet_3.pdf]

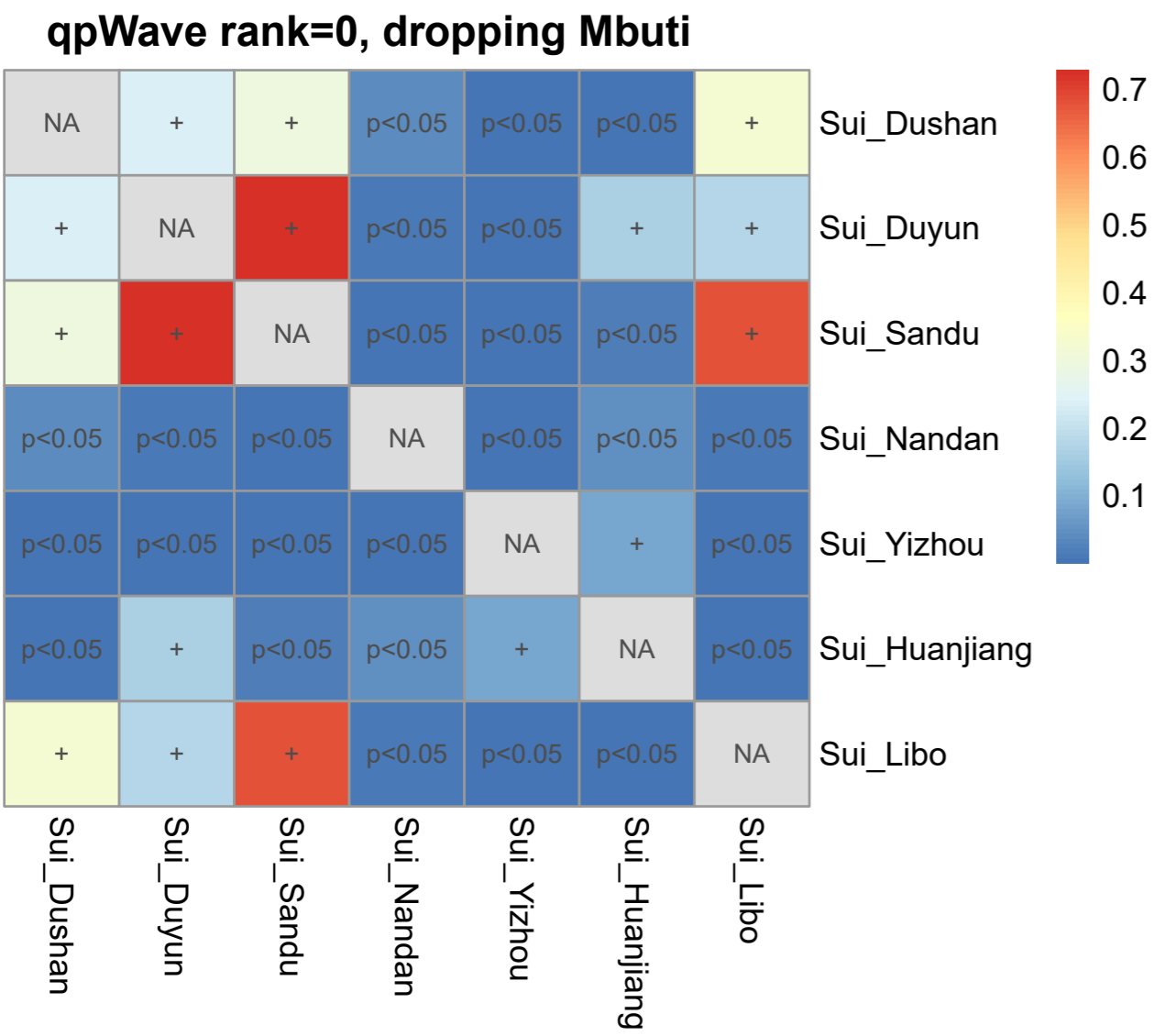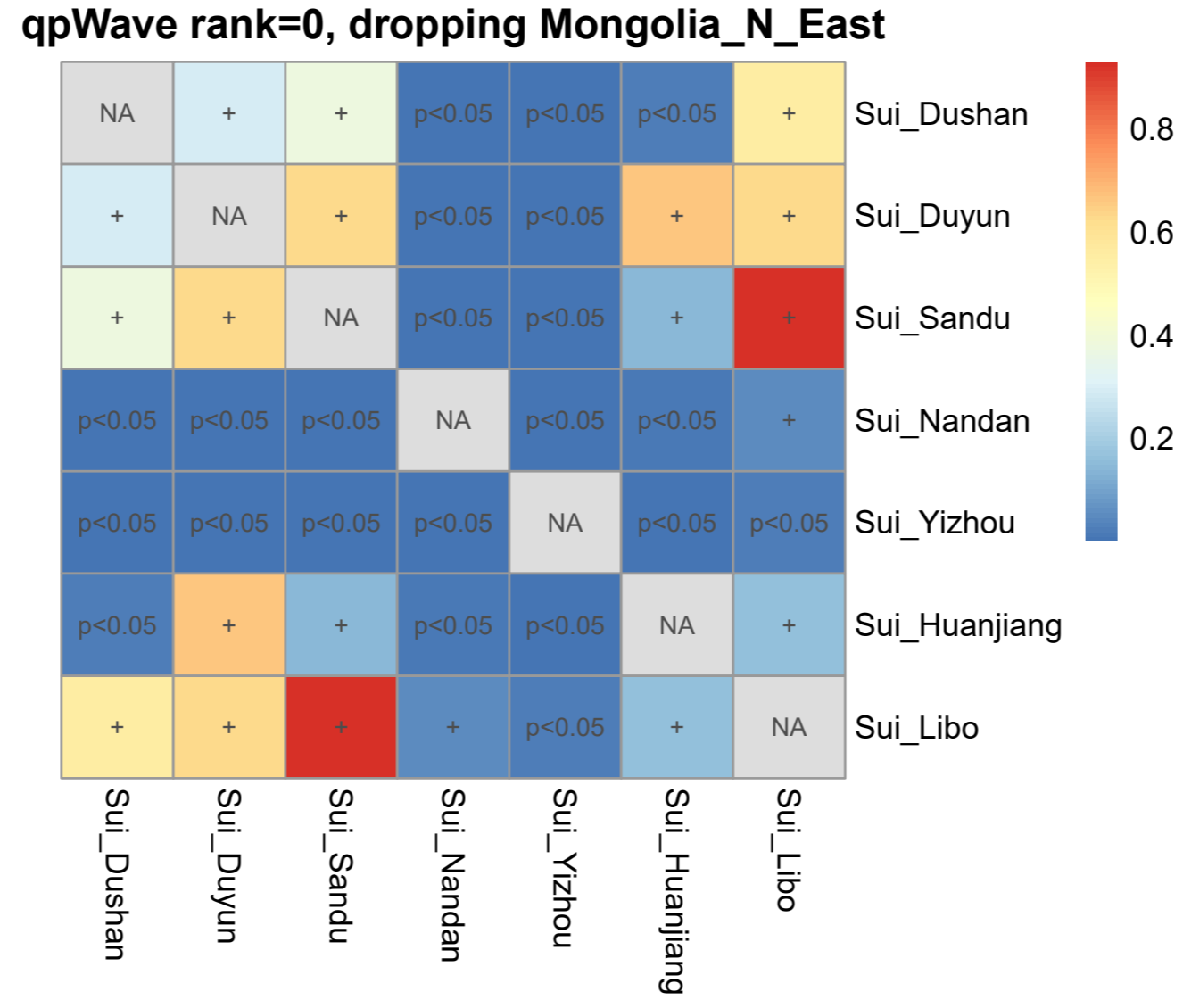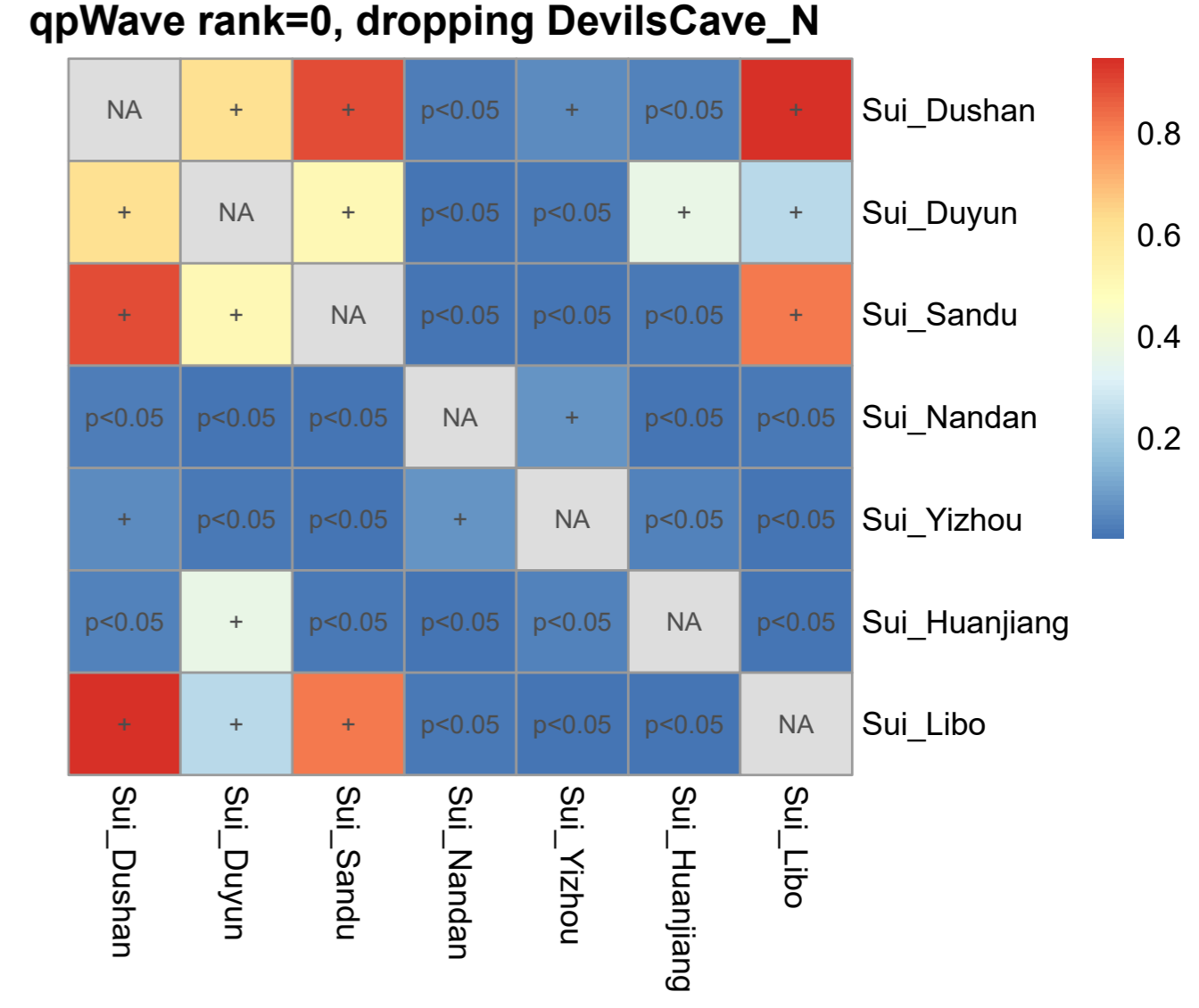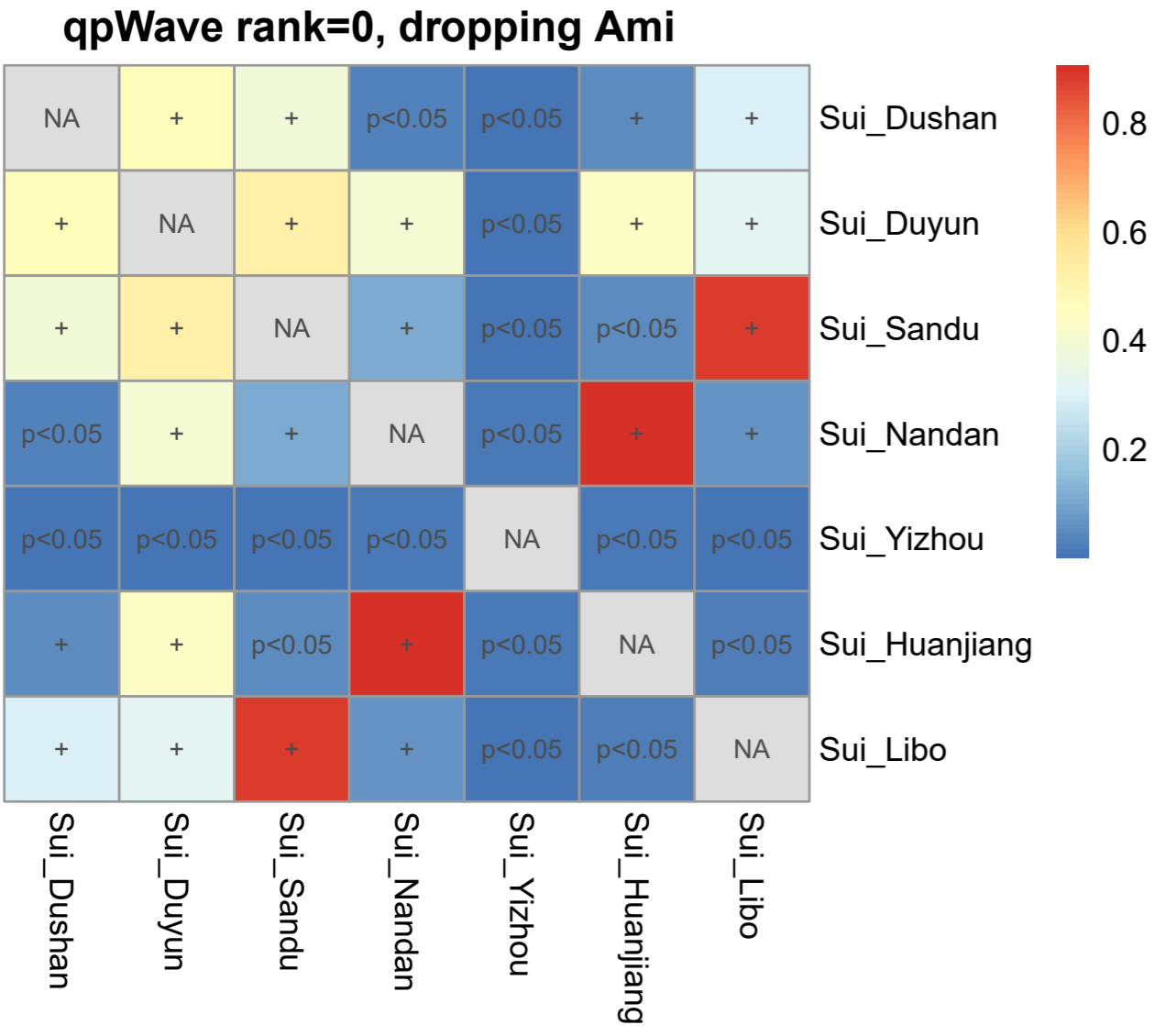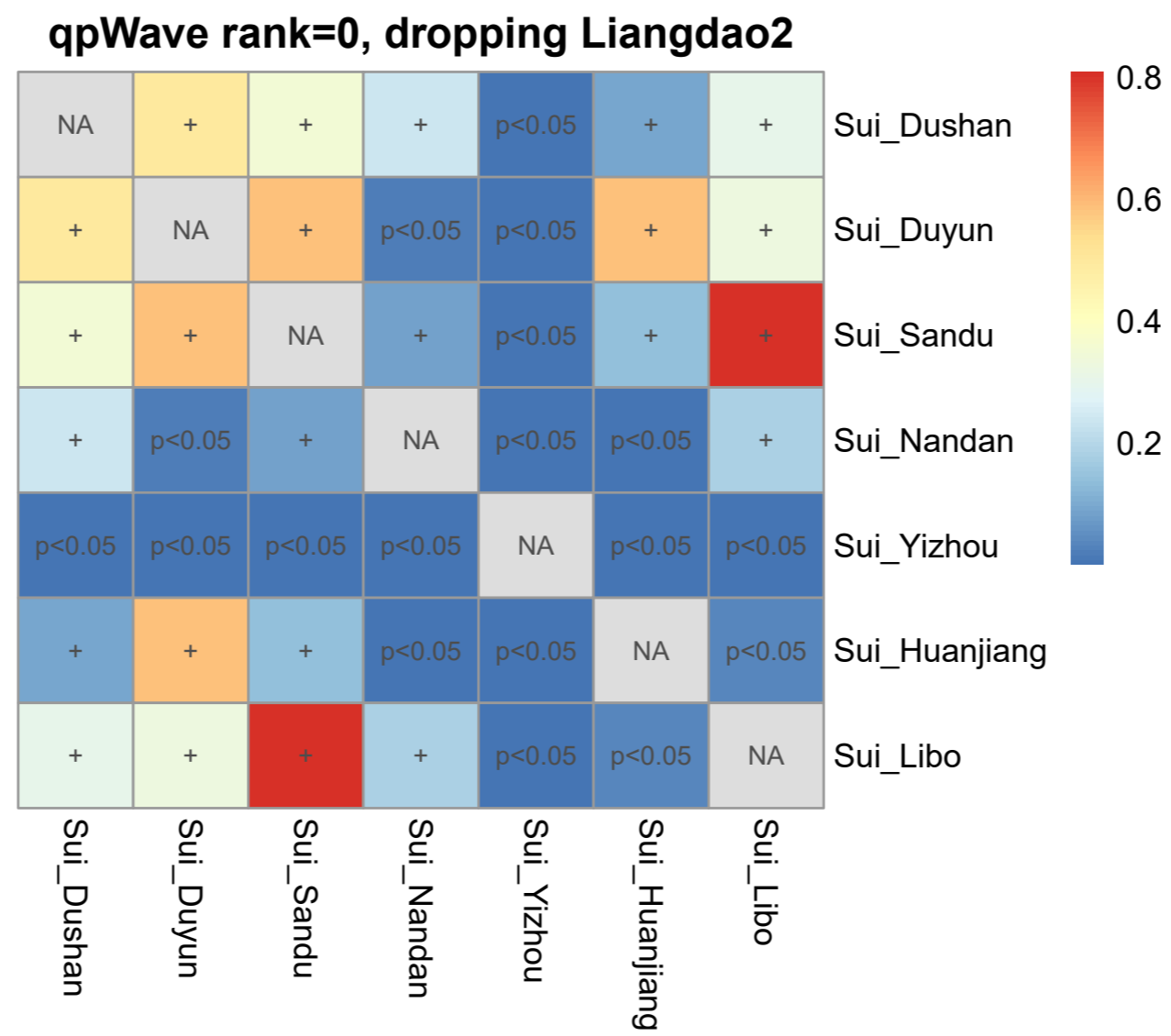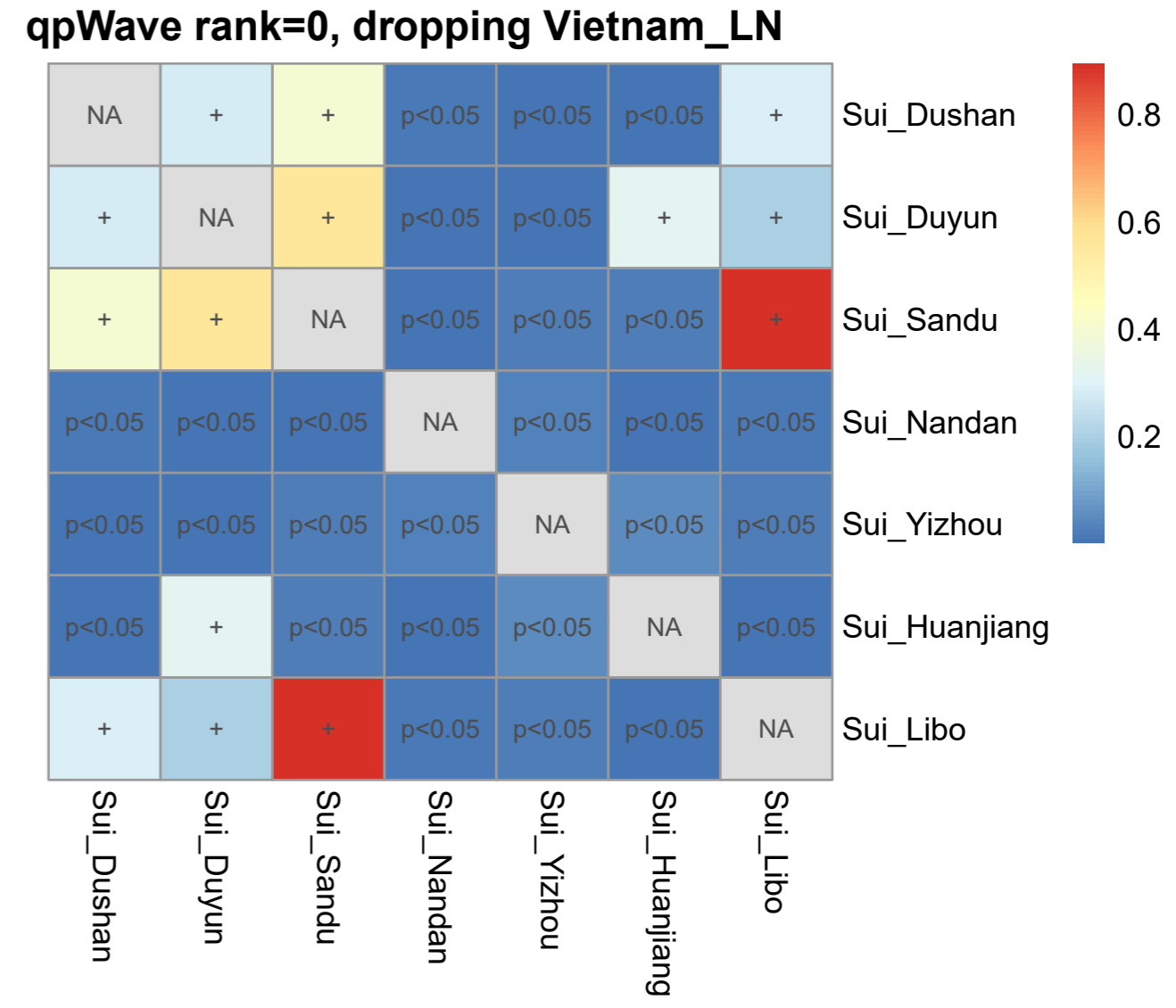

Supplement: Supplementary Figure 4 — qpWave Outgroup dropping test, in which we dropped one of the populations in the outgroup set by turn (Mbuti, Mongolia_N_East, DevilsCave_N, Ami, Liangdao2, Vietnam_LN). If we observed P > 0.05 for rank = 0, while P < 0.05 (rank = 0) in the no-drop qpWave test, suggesting the dropped population might have a unique gene flow with one of the test groups, explained the non-homogeneity between the pairwise test populations. [file Data_Sheet_4.pdf]

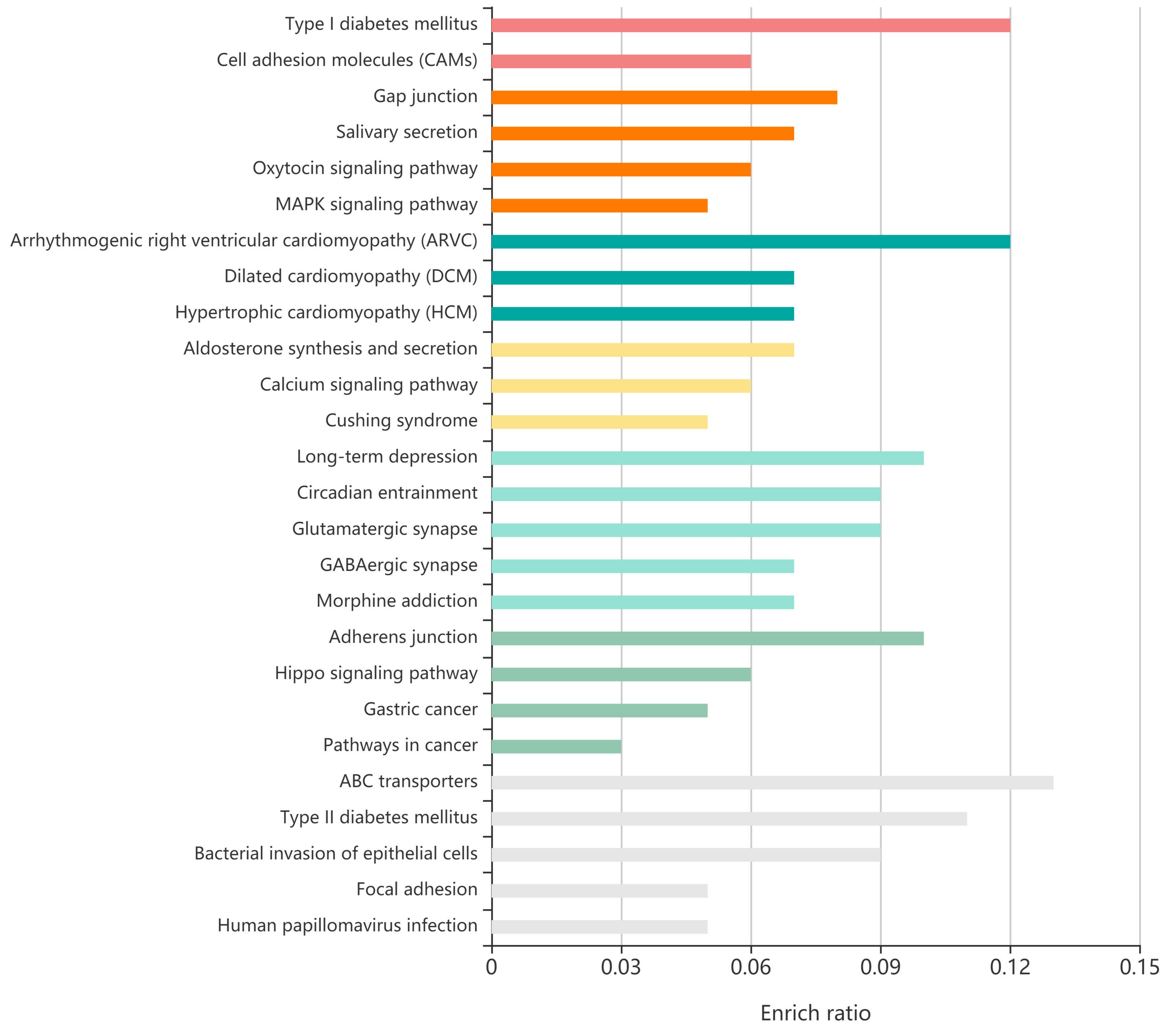

Supplement: Supplementary Figure 5 — KEGG pathway analysis. Each row represented an enriched function, and the length of the bar represented the enrich ratio which was calculated as “input gene number”/“background gene number.” The color of the bar represents different clusters. [file Data_Sheet_5.pdf]
